# Supplementary material for: The integration of metabolic and proteomic data uncovers an augmentation of the sphingolipid biosynthesis pathway during T-cell differentiation
Source: Commun Biol. 2024 May 23;7:622. doi: 10.1038/s42003-024-06339-7 (PMC11116545; doi:10.1038/s42003-024-06339-7)
Supplement: Supplementary file 2 — Supplementary Information [file 42003_2024_6339_MOESM2_ESM.pdf]

Supplementary Figure 1

a

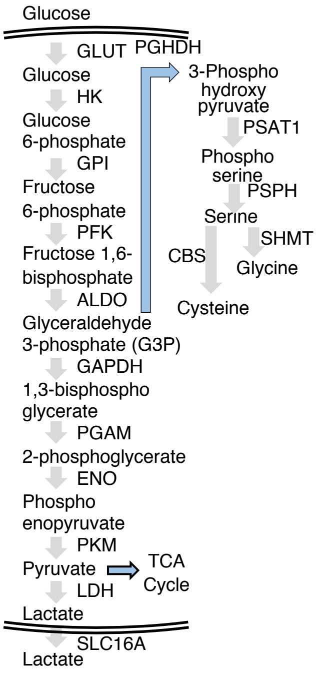

b

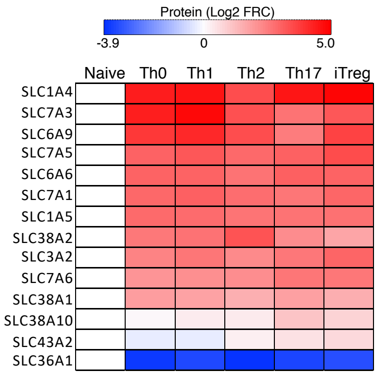

Supplementary Figure 2

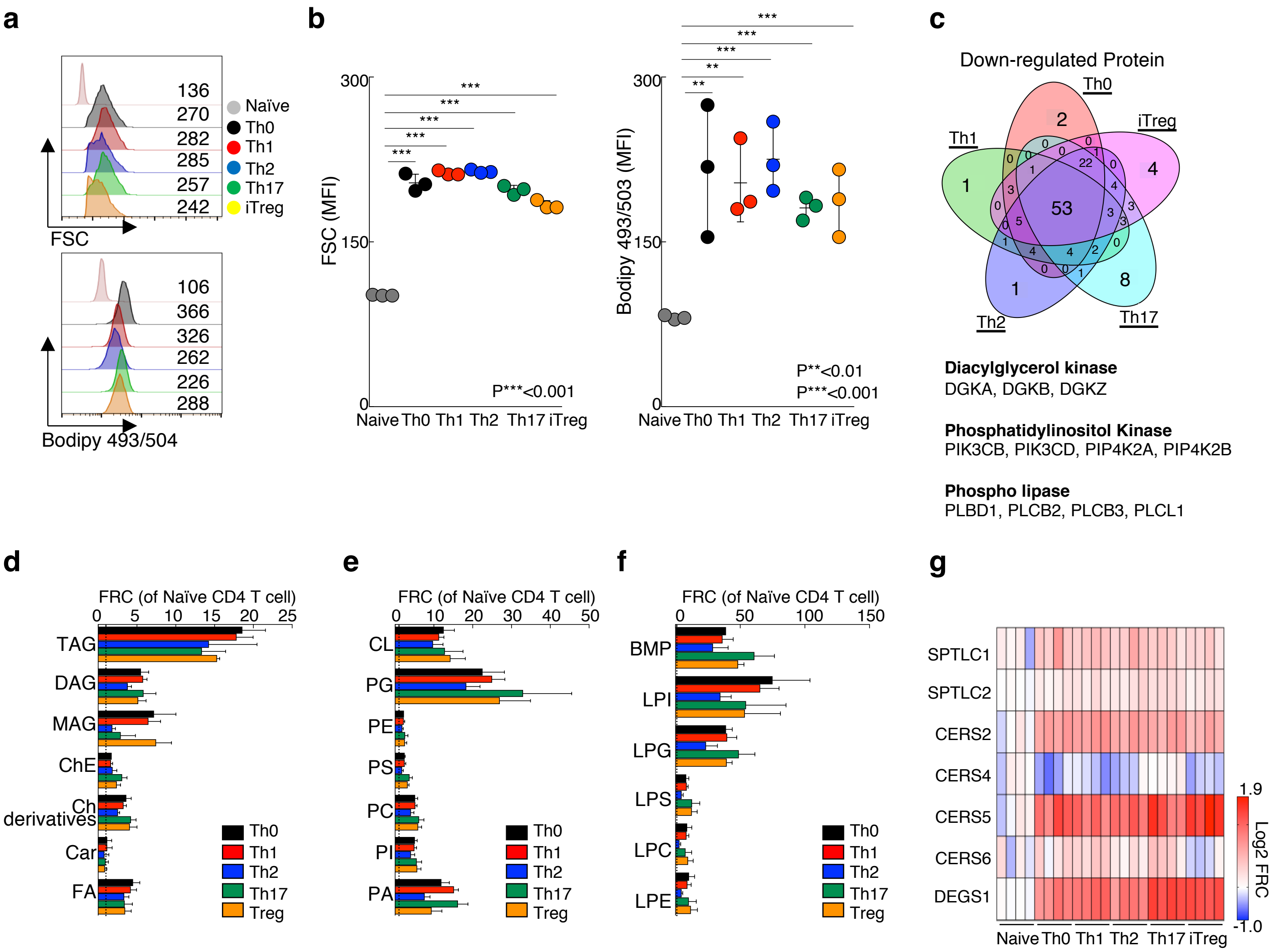

Supplementary Figure 3

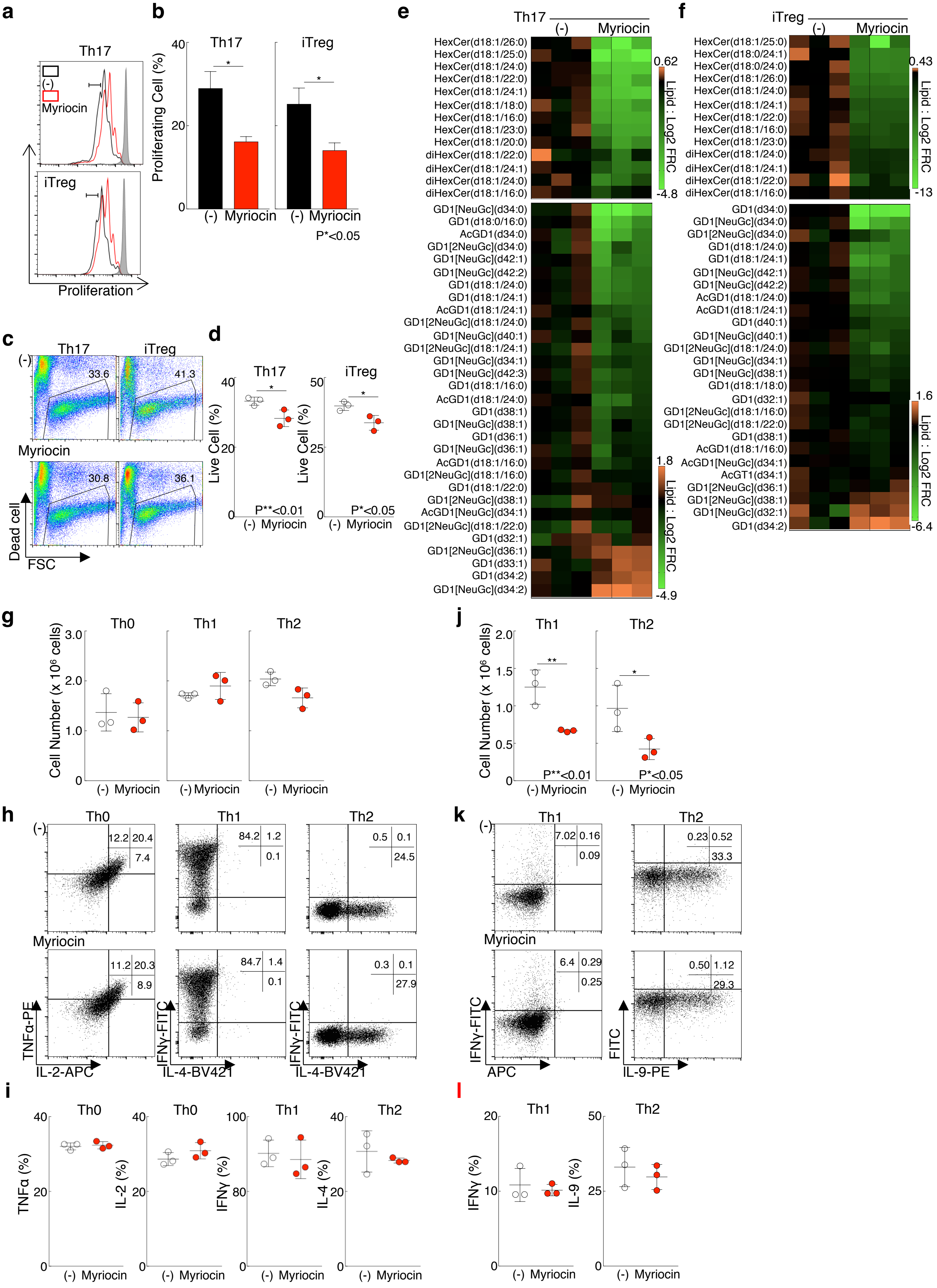

Supplementary Figure 4

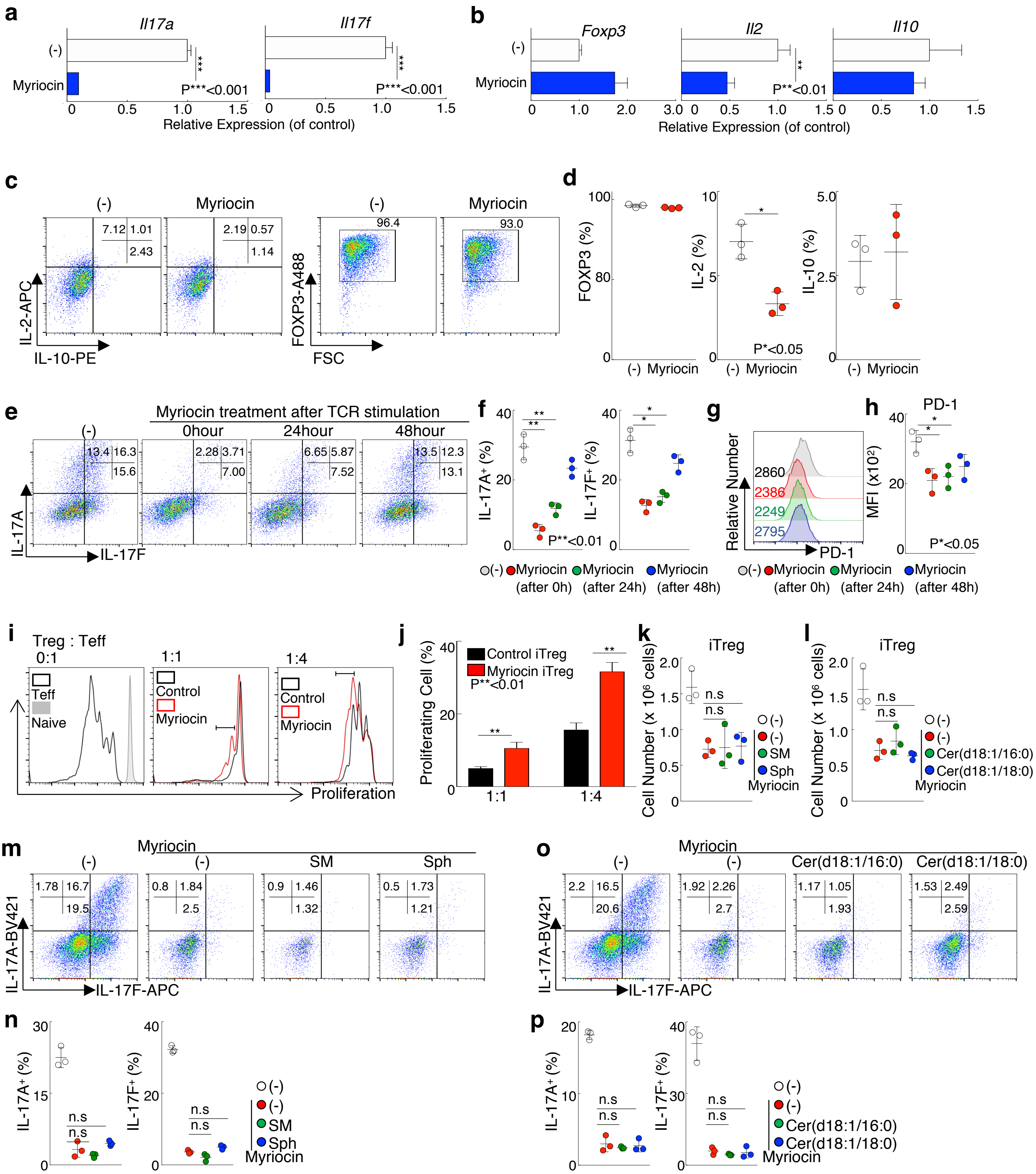

Supplementary Figure 5

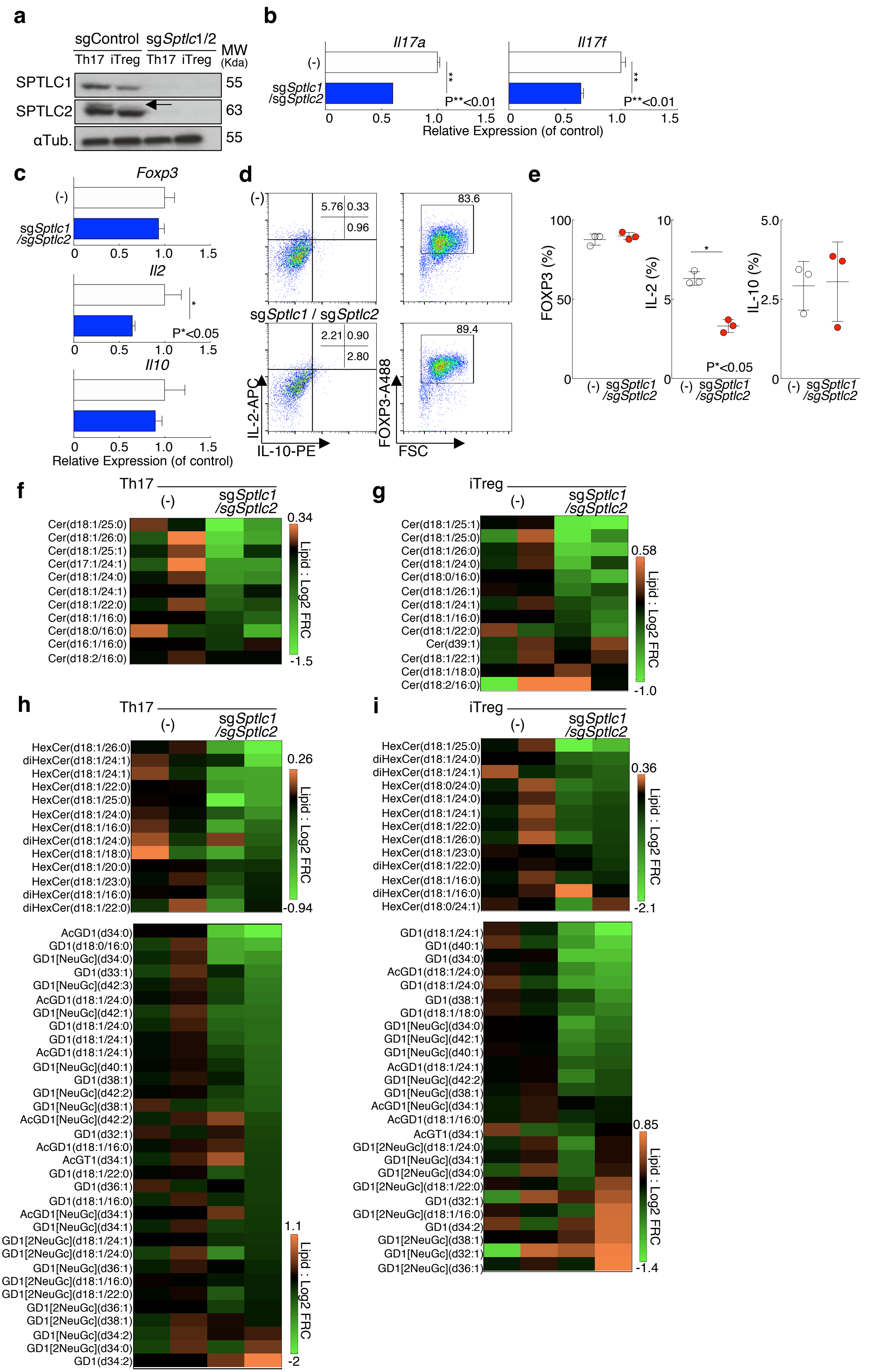

Supplementary Figure 6

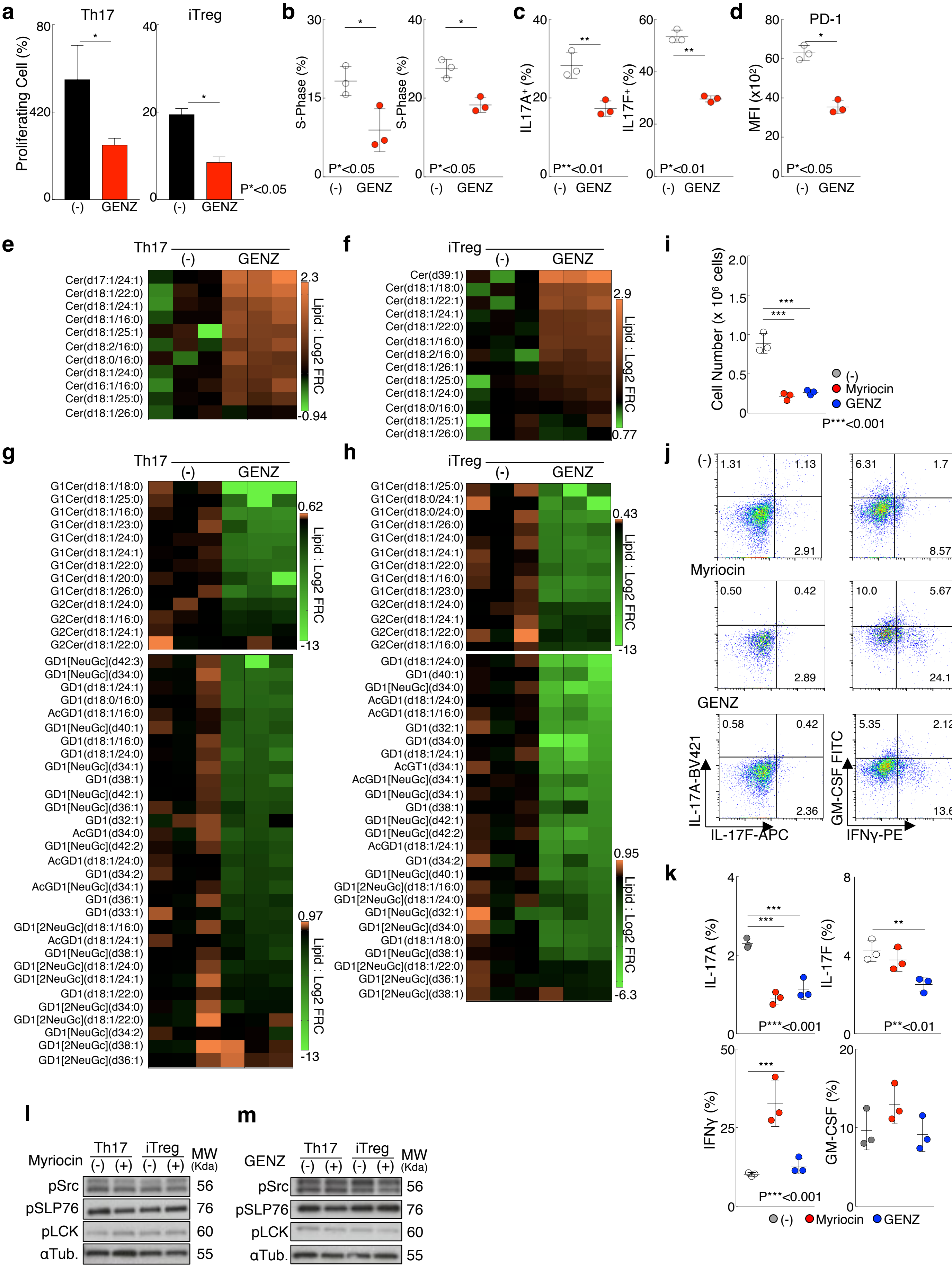

Supplemental Information

Supplementary Figure

Supplementary Fig.1 Proteomics anaysis revealed significant increases in the expression of amino acid transporters.

a, Overview of the serine biosynthesis pathway. **b**, Heatmap depict the protein expression of amino acid transporters. Four biological replicate was prepared for proteomics analysis.

Supplementary Fig.2 T-cell activation dynamically changes lipid metabolism accompanied by the changes in the protein expression related to lipid metabolism.

a, b, FACS analysis shows FSC and bodipy 493/503 of each Th cell subsets (a). Summarized data was shown here (b). **c**, Venn diagram showed overlaps and differences between 2.0-fold decreased proteins related to lipid metabolism in Th0, Th1, Th2, Th17, or iTregs cells relative to naive $CD4^{+}$T cells. **d-f**, The lipidomics analysis shows the relative contents of lipid species related to glycerolipids, cholesterol, and free fatty acids (**d**), or phospholipids (**e**), or lysophospholipids (**f**) in Th0, Th1, Th2, Th17, and iTreg cells compared to naïve $CD4^{+}$ T cells. **g**, Heatmap depict the protein expression related to ceramide biosynthesis. Three independent experiments were summarized (**a**,**b**). Four biological replicate was prepared for metabolomics and proteomics analysis.

Supplementary Fig.3 Inhibition of sphingolipid metabolism failed to suppress the generation of Th0, Th1, and Th2 cells.

a, Naive $CD4^+$ T cells were labelled with e670 proliferation dye and stimulated with immobilized anti-TCRβ mAb and anti-CD28 mAb in the presence of myriocin under Th17 or iTreg polarization condition. **b,** The division number of cell related to (**a**) was shown. **c**, Effects of myriocin on cell survival was evaluated by FACS analysis. **d**, Summarized data related to (**c**) was shown. **e**, **f,** Heatmap depict the levels of glycosphingolipid in myriocin treated-Th17 (**e**) and iTreg cells (**f**). **g**, Cell number of Th0, Th1, or Th2 cells treated with DMSO or 6.6μM myriocin was shown. **h,** Intracellular staining of TNFα and IL-2 in Th0 cells or IFNγ and IL-4 in Th1 and Th2 cells treated with myriocin was shown. **i,** Summary data of TNFα, IL-2, IFNγ or IL-4 expression related to (**h**) was shown. **j**, Cell number of TGFβ-treated Th1, or Th2 cells treated with DMSO or 6.25μM myriocin was shown. **k,** Intracellular staining of IFNγ in Th1 or IL-9 in Th2 cells treated with myriocin in the presence of TGFβ was shown. **l,** Summary data of IFNγ or IL-9 expression related to (**k**) was shown. Relative expression (normalized to Hprt) with SD is shown. Three independent experiments were performed and showed similar results (**a-d, g-l**). Three biological replicate was prepared for metabolomics and proteomics analysis.

Supplementary Fig.4 Sphingolipid metabolism were required to regulate Th17, and iTreg cells differentiation.

a,b, qRT-PCR analyses of the relative expression of *Il17a* and *Il17f* **(a)** in myriocin-treated Th17 cells or *Foxp3, Il2, or Il10* **(b)** in myriocin-treated iTreg cells. **c**, FACS analysis showed that protein expression of FOXP3, IL-2, and IL-10 in myriocin-treated iTreg cells. **d**, Summary data of FOXP3, IL-2, and IL-10 expression related to (**c**) was shown. **e-h**, FACS analysis shows IL-17A and IL-17F production of Th17 cells **(e)** or PD-1 expression **(g)** in iTreg cells treated with myriocin after 0, 24, or 48 hours TCR stimulation. Summary data of protein expression related to IL-17A and IL-17F production (**f**), or PD-1 expression **(h)** was shown. **i**, FACS analysis showed suppressive capacity of myriocin-treated iTreg cells. **j**, Summary data of suppressive capacity related to (**i**) was shown. **k**, **l**, Summarized data shows cell number of iTreg cells treated with 2μM SM or 2μM Sph (**k**) or 0.2μM Cer(d18:1/16:0) or 0.2μM Cer(d18:1/18:0) (**l**) in the presence of 6.6μM myriocin. **m-p**, FACS analysis shows IL-17A and IL-17F production of Th17 cells treated with 2μM SM or 2μM Sph (**m**), or 0.2μM Cer(d18:1/16:0) or 0.2μM Cer(d18:1/18:0) (**o**) in the presence of 6.6μM myriocin. Summarized data was shown related to Supplementary Fig. 4m **(n)** and 4o **(p)**. Three independent experiments were performed and showed similar results (**c-p**). Three technical replicates were performed with quantitative RT-PCR (**a,b**). Error bar indicates SD. Three biological replicate was prepared for metabolomics analysis.

Supplementary Fig.5 gene deletion of *Sptlc1/2* inhibited differentiation of naïve CD4+ T cells into Th17, and iTreg cells.

a, WB analysis evaluated protein expression of SPTLC1 and SPTLC2 in control or sg*Sptlc1/2* Th17 and iTreg cells. **b, c,** qRT-PCR analyses of the relative expression of *Il17a* and *Il17f* (**b**) in sg*Sptlc1/2* Th17 cells or *Foxp3, Il2, or Il10* (**c**) in sg*Sptlc1/2* iTreg cells. **d**, FACS analysis showed that protein expression of FOXP3, IL-2, and IL-10 in sg*Sptlc1/2* iTreg cells. **e**, Summary data of FOXP3, IL-2, and IL-10 expression related to (**d**) was shown. **f-i** Heatmap depict the levels of sphingolipid or glycosphingolipid in sg*Sptlc1/2* Th17 (**f,h**) and iTreg cells (**g,i**). Three independent experiments were performed and showed similar results (**c-e**). Three technical replicates were performed with quantitative RT-PCR (**a,b**). Error bar indicates SD. Two biological replicate was prepared for metabolomics.

Supplementary Fig.6 Inhibition of glycosphingolipid metabolism suppressed the generation of Th17 and Treg cells.

a, **b**, Summary data of cell division (**a**) or cell cycle (**b**) related to (Fig. 5j, k) was shown. **c**, **d**, Summary data of IL-17A, IL17F (**c**), or PD-1(**d**) expression related to (**Fig. 5l**) or (**Fig. 5m**) was shown. **e-h** Heatmap depict the levels of sphingolipid or glycosphingolipid in GENZ-123346 treated Th17 (**e,g**) and iTreg cells (**f,h**). **i**, Cell number of pathogenic Th17 cells treated with DMSO, myriocin, or GENZ-123346 was shown. **j**,

FACS analysis showed that protein expression of IL-17A, IL-17F, GM-CSF, and IFNγ in pathogenic Th17 cells treated with DMSO, myriocin, or GENZ-123346. **k**, Summary data of IL-17A, UL-17F, GM-CSF, and IFNγ expression related to (**j**) was shown. **l, m,** WB analysis evaluated phosphorylation levels expression of Src, SLP76, and Lck in DMSO, myriocin **(l)**, or GENZ-123346 **(m)** treated with Th17 and iTreg cells. Three independent experiments were performed and showed similar results (**a-d, i-m**). Error bar indicates SD. Three biological replicate was prepared for metabolomics.
