# Supplementary material for: The integration of metabolic and proteomic data uncovers an augmentation of the sphingolipid biosynthesis pathway during T-cell differentiation
Source: Commun Biol. 2024 May 23;7:622. doi: 10.1038/s42003-024-06339-7 (PMC11116545; doi:10.1038/s42003-024-06339-7)
Supplement: Supplementary file 3 — Description of additional supplemental files [file 42003_2024_6339_MOESM3_ESM.docx]

Description of Additional Supplemental Files

File name: Supplementary Data 1

Description: The source data and figure data of the metabolomics and lipidomics data

File name: Supplementary Data 2

Description: The source data behind the graphs.
