# Supplementary material for: The integration of metabolic and proteomic data uncovers an augmentation of the sphingolipid biosynthesis pathway during T-cell differentiation
Source: Commun Biol. 2024 May 23;7:622. doi: 10.1038/s42003-024-06339-7 (PMC11116545; doi:10.1038/s42003-024-06339-7)
Supplement: Supplementary file 6 — Reporting Summary [file 42003_2024_6339_MOESM6_ESM.pdf]

nature portfolio

Corresponding author(s): Marianne Stoessel

Last updated by author(s): May 7, 2024

Reporting Summary

Nature Portfolio wishes to improve the reproducibility of the work that we publish. This form provides structure for consistency and transparency in reporting. For further information on Nature Portfolio policies, see our Editorial Policies and the Editorial Policy Checklist.

Statistics

For all statistical analyses, confirm that the following items are present in the figure legend, table legend, main text, or Methods section.

n/a	Confirmed	
☐	☒	The exact sample size (*n*) for each experimental group/condition, given as a discrete number and unit of measurement
☐	☒	A statement on whether measurements were taken from distinct samples or whether the same sample was measured repeatedly
☐	☒	The statistical test(s) used AND whether they are one- or two-sided<br>*Only common tests should be described solely by name; describe more complex techniques in the Methods section.*
☐	☒	A description of all covariates tested
☐	☒	A description of any assumptions or corrections, such as tests of normality and adjustment for multiple comparisons
☐	☒	A full description of the statistical parameters including central tendency (e.g. means) or other basic estimates (e.g. regression coefficient) AND variation (e.g. standard deviation) or associated estimates of uncertainty (e.g. confidence intervals)
☒	☐	For null hypothesis testing, the test statistic (e.g. *F*, *t*, *r*) with confidence intervals, effect sizes, degrees of freedom and *P* value noted<br>*Give P values as exact values whenever suitable.*
☒	☐	For Bayesian analysis, information on the choice of priors and Markov chain Monte Carlo settings
☒	☐	For hierarchical and complex designs, identification of the appropriate level for tests and full reporting of outcomes
☒	☐	Estimates of effect sizes (e.g. Cohen's *d*, Pearson's *r*), indicating how they were calculated

Our web collection on statistics for biologists contains articles on many of the points above.

Software and code

Policy information about availability of computer code

Data collection	The acceleration data was collected using the TechnoSmart software, while the GPS data was collected using their respective brand software (Followit, FindMy or TechnoSmart).
Data analysis	All analyses were performed on R version 4.0 and later.

For manuscripts utilizing custom algorithms or software that are central to the research but not yet described in published literature, software must be made available to editors and reviewers. We strongly encourage code deposition in a community repository (e.g. GitHub). See the Nature Portfolio guidelines for submitting code & software for further information.

Data

Policy information about availability of data

All manuscripts must include a data availability statement. This statement should provide the following information, where applicable:

- Accession codes, unique identifiers, or web links for publicly available datasets
- A description of any restrictions on data availability
- For clinical datasets or third party data, please ensure that the statement adheres to our policy

The authors confirm that if the article should be accepted, the data supporting the results, together with the R 'source code' used for the analysis, will be archived in the public repository Figshare (link: https://figshare.com/s/3e63b25c5a776196a69f). A DOI number for the data repository will be also included in the article.

Research involving human participants, their data, or biological material

Policy information about studies with human participants or human data. See also policy information about sex, gender (identity/presentation), and sexual orientation and race, ethnicity and racism.

Reporting on sex and gender	NA
Reporting on race, ethnicity, or other socially relevant groupings	NA
Population characteristics	NA
Recruitment	NA
Ethics oversight	NA

Note that full information on the approval of the study protocol must also be provided in the manuscript.

Field-specific reporting

Please select the one below that is the best fit for your research. If you are not sure, read the appropriate sections before making your selection.

☐ Life sciences ☐ Behavioural & social sciences ☒ Ecological, evolutionary & environmental sciences

For a reference copy of the document with all sections, see nature.com/documents/nr-reporting-summary-flat.pdf

Ecological, evolutionary & environmental sciences study design

All studies must disclose on these points even when the disclosure is negative.

Study description	Monitoring of reindeer activities using acceleration data loggers in relationship with temperature (recorded from the same data loggers, and from the nearest weather stations).
Research sample	31 reindeer from three Swedish reindeer districts monitored in summer 2019 and 2020.
Sampling strategy	Sampling strategy consisted of monitoring 10 female reindeer per district per year, i.e. 30 reindeer in summer 2019 and 30 in 2020 as well.
Data collection	Data collection was done in agreement and full collaboration of the reindeer herders of the three districts of Handölsdalen, Gran and Sirges. The herders themselves gathered the reindeer and tagged them with the data loggers. They also captured the reindeer and retrieved the collars themselves the following autumn.
Timing and spatial scale	The reindeer were collared with the data loggers for calf-marking (around start of July/mid-July) by the herders and the data loggers were collected in the autumn when the herders gather their reindeer for slaughtering (around October). Exact dates are given in the supplementary material Table S1
Data exclusions	Data was excluded when the reindeer were still herded, and hence their activities were disturbed by human presence. This type of data was considered outlier and represented 2.46% of the total GPS data.
Reproducibility	The monitoring took place two years in a row (summer 2019 and 2020), in order to repeat the monitoring and verify the findings from the 1st monitoring year.
Randomization	The reindeer monitored were female randomly chosen by the herders
Blinding	NA

Did the study involve field work? ☒ Yes ☐ No

Field work, collection and transport

Field conditions	Clear and calm summer conditions in the Swedish mountains with no strong wind, nor precipitation. The timing was chosen by the reindeer herders to implement their calf-marking.
Location	The reindeer were tagged by the herders of the respective reindeer herding districts Sirges (Norrbotten county, 67°00′N 17°00′E), Gran (Västerbotten county, 66°00′N 16°00′E) and Handölsdalen (Jämtland county, 63°00′N 12°00′E).

Access & import/export	As the reindeer tagged were livestock, and the handling of the reindeer was done by the reindeer herders who owned the animals, this study did not require a specific ethical permit according to The Swedish Board of Agriculture (Statens jordbruksverks föreskrifter och allmänna råd om försöksdjur SJVFS 2019:9.
Disturbance	Capturing the reindeer to put collars on them was a disturbance, which we minimised by agreeing that the herders would do it when they gather their animals for calf-marking. The total weight of the collars were also less than 2% of the total reindeer body mass.

Reporting for specific materials, systems and methods

We require information from authors about some types of materials, experimental systems and methods used in many studies. Here, indicate whether each material, system or method listed is relevant to your study. If you are not sure if a list item applies to your research, read the appropriate section before selecting a response.

Materials & experimental systems

n/a	Involved in the study	
☒	☐	Antibodies
☒	☐	Eukaryotic cell lines
☒	☐	Palaeontology and archaeology
☐	☒	Animals and other organisms
☒	☐	Clinical data
☒	☐	Dual use research of concern
☒	☐	Plants

Methods

n/a	Involved in the study	
☒	☐	ChIP-seq
☒	☐	Flow cytometry
☒	☐	MRI-based neuroimaging

Animals and other research organisms

Policy information about studies involving animals; ARRIVE guidelines recommended for reporting animal research, and Sex and Gender in Research

Laboratory animals	The study did not involve laboratory animals
Wild animals	The study did not involve wild animals, but involve livestock reindeer, owned by herders.
Reporting on sex	We monitored female reindeer, and no male.
Field-collected samples	N/A
Ethics oversight	No ethical permit was necessary to do the monitoring (according to Swedish law Statens jordbruksverks föreskrifter och allmänna råd om försöksdjur SJVFS 2019:9), because we had the agreement of the owners of the animals who tagged their livestock themselves.

Note that full information on the approval of the study protocol must also be provided in the manuscript.

Plants

Seed stocks	NA
Novel plant genotypes	NA
Authentication	NA
